# Update on antimicrobial peptides: key elements in Orthoflavivirus infection – an overview

**DOI:** 10.1099/jgv.0.002129

**Published:** 2025-07-03

**Authors:** Victor Javier Cruz-Holguín, Ivonne Sagrario Romero-Flores, Luis Gerardo Olmos-Bustos, Julio García-Cordero, Moisés León-Juárez, Leticia Cedillo-Barron

**Affiliations:** 1 Departamento de Biomedicina Molecular, Centro de investigación y Estudios avanzados del IPN (CINVESTAV), Av Instituto Politécnico Nacional 2508, San Pedro Zacatenco, Gustavo A. Madero, 07360, Ciudad de México, Mexico; 2 Laboratorio de Virología Perinatal y Diseño Molecular de Antígenos y Biomarcadores, Departamento de Inmunobioquimica, Instituto Nacional de Perinatología, C. Montes Urales 800, Lomas - Virreyes, Lomas de Chapultepec IV Sec, Miguel Hidalgo, 11000 Ciudad de México, Mexico; 3 Departamento de Genética y Biología Molecular, Centro de investigación y Estudios avanzados del IPN (CINVESTAV), Av Instituto Politécnico Nacional 2508, San Pedro Zacatenco, Gustavo A. Madero, 07360 Ciudad de México, Mexico

**Keywords:** antimicrobial, antiviral, immunoregulation, *Orthoflaviviruses*, peptides

## Abstract

*Flaviviridae* is a family of viruses that are mainly transmitted by mosquito vectors of the genus *Aedes*, which cause febrile illnesses and, in severe cases, haemorrhagic or neurodegenerative conditions. Over time, these viruses have been reported as emerging pathogens, leading to epidemic outbreaks in various regions worldwide. Additionally, climate change has facilitated the migration of these vectors to regions where they were not previously found. Unfortunately, there are currently no effective treatments or vaccines to prevent or combat *Orthoflavivirus* infections. Consequently, a deeper understanding of the viral biology and the human host immune response is crucial for advancing the development of therapeutic targets. Amongst the molecules involved in the innate immune response to viral infections are antimicrobial peptides (AMPs), which have been studied for decades. However, their role in *Orthoflavivirus* infections remains poorly understood. Several researchers have proposed the stimulation or exogenous administration of AMPs during various viral infections, highlighting these molecules as potential innovative therapeutic targets. This study compiles current knowledge on AMPs with a specific focus on *Orthoflavivirus* infections, emphasizing the importance of these promising therapeutic approaches.

## Introduction

Antimicrobial peptides (AMPs) are small amino acid sequences initially described for their antibacterial properties [1]. However, in recent years, they have been described to exert effects against fungi, bacteria and, notably, viruses. The antiviral effects of AMPs have been widely described for viral infections such as human immunodeficiency virus (HIV), herpes simplex virus (HSV) and influenza virus (IVA) [2,3]. Despite this, the role of AMPs in combating viruses from the *Flaviviridae* family has been poorly characterized. Therefore, this review aims to compile the most current information regarding the effect of AMPs in the context of *Orthoflavivirus* infections.

The *Flaviviridae* family consists of viruses primarily transmitted by two types of vectors: mosquitoes, including *Aedes albopictus* (*Ae. Albopictus*) and *Aedes aegypti* (*Ae. Aegypti*) [4,5], and ticks, such as *Ixodes ricinus*, *Ixodes persulcatus* and *Ixodes percatus* [6]. Certain viruses, such as Zika virus (ZIKV), can also be transmitted sexually and vertically (from mother to fetus). These transmission routes involve histological sites rich in epithelial cells and immune system infiltration, which are key in AMP production [7]. The *Flaviviridae* family comprises four genera: *Pegivirus*, *Hepacivirus*, *Pestivirus* and *Orthoflavivirus* (formerly *Flavivirus*, which are of interest in this article). They are transmitted by arthropod-borne viruses (arboviruses) such as mosquitoes and ticks, Powassan virus and tick-borne encephalitis virus (TBEV) amongst others and mosquito-borne viruses such as ZIKV, dengue virus (DENV) and West Nile virus (WNV), for example [4,5, 8, 9].

Orthoflaviviruses possess a single-stranded positive-sense RNA genome containing a single ORF that encodes a viral polyprotein. This polyprotein undergoes proteolytic processing to yield seven nonstructural proteins (NS1, NS2A, NS2B, NS3, NS4A, NS4B and NS5) and three structural proteins (E, C and M) [4]. Their genome also includes two highly structured untranslated regions at the 5′ and 3′ ends. Nucleolytic processing of genomic orthoflaviviral RNA gives rise to three to four subgenomic RNAs Subgenomic flaviviral RNAs(sfRNA) which are involved in immune evasion as well [10,11]. Infections caused by these viruses are typically characterized by mild to moderate clinical manifestations, such as mild fever and joint pain. However, some cases present severe symptoms, including haemorrhagic fever. Additionally, certain flaviviruses, such as Japanese encephalitis virus, ZIKV, WNV and TBEV, can cause central nervous system (CNS) damage [12].

During the transmission of these viruses by their vectors, it is important to consider that the immune response of the vector plays an important role in regulating the viral load. Interestingly, vector and human AMPs are highly similar in their protein structure and antimicrobial mechanisms, as they are highly evolutionarily conserved molecules. In general, arthropod AMPs have been classified as defensins, cecropins, attacines and drosomysins, structurally composed of small sequences of 20–50 aa, with structures rich in alpha helices and beta sheets stabilized by disulphide bonds (similar to human AMPs), and some can adopt linear or cyclic structures (similar to θ-defensins of non-human primates) [13,14].

AMPs from the mosquito vector *Ae. aegypti* have demonstrated antiviral activity during the transmission of some arboviruses, as observed during infection by some *Orthoflaviviridae*. Different cell lines of the *Aedes* mosquito produce defensin C, which has a restrictive effect during DENV infection [15]. Furthermore, the presence of different AMPs in the saliva of mosquito vectors has been detected, such as AAEL000598 and cecropin, both produced in the salivary glands. Cecropin has demonstrated antiviral activity against DENV [16]. Mosquito C6/36 cells have also been shown to respond to DENV infection by producing AMPs, such as defensins C, E and A [17].

In humans, AMPs are mainly produced by epithelial cells and cells of the immune system; on the other hand, in ticks and mosquitoes, AMPs are mainly produced by different types of cells in the midgut and, mainly, by the salivary glands, since the immune system of the mosquito vector is mainly governed by the innate immune system, whilst in mammals such as humans, they have both an innate and adaptive immune system [14,18]. Therefore, arthropod AMPs are a fundamental tool for their defence, although, in humans, they are also important, but they are not the only defence barrier. Some authors suggest that the function of these AMPs could be to control viral spread in the vector [18]. However, viruses manage to overcome this immune barrier and can be transmitted to their human host. In this context, human AMPs have also been shown to have an antiviral effect on several viruses, including *Orthoflaviviridae*. However, like other viruses, *Orthoflaviviridae* possess evasion mechanisms that allow them to survive and replicate in their mammalian host.

These viruses are also prone to mutations, since the RNA-dependent RNA polymerase has low fidelity. This could generate errors and accumulate various mutations. These, in turn, theoretically could result in the production of various changes in the epitopes that could evade recognition by neutralizing antibodies. For example, it has been reported that there are mutations in domain II of the E protein of the WNV, DENV, yellow fever virus (YFV) and TBEV viruses, where these mutations can escape neutralizing antibodies [19,23]. The use of drugs to combat *Orthoflavivirus* infections remains limited, and the application of vaccines and pharmaceutical treatments is constrained. Human AMPs are promising therapeutic agents due to their low cytotoxicity, absence of reported pathogen resistance and multiple mechanisms of action. Despite their potential, the mechanisms of action of AMPs during *Orthoflavivirus* infections are not well understood. Interest in these peptides has grown in the past decade, but limited information is available. Therefore, this study compiles recent advances in AMP research, with a specific focus on orthoflaviviral infections.

## General features of AMPs

AMPs are short cationic amino acid sequences (10–50 amino acids) with amphipathic properties. These peptides are produced by a wide variety of organisms, including bacteria, fungi, insects, plants, amphibians and mammals such as mice, monkeys and humans [24]. According to the AMP database from the University of Nebraska, over 4,000 AMPs have been registered as of 2024, originating from both synthetic and natural sources across various species. This review focuses on AMPs derived from human sources, such as defensins, cathelicidins (LL-37) and peptides containing the whey acidic protein (WAP) domain [trappin-2/elafin (Tr2/E)] and secretory leucocyte protease inhibitor (SLPI).

**Fig. 1.**
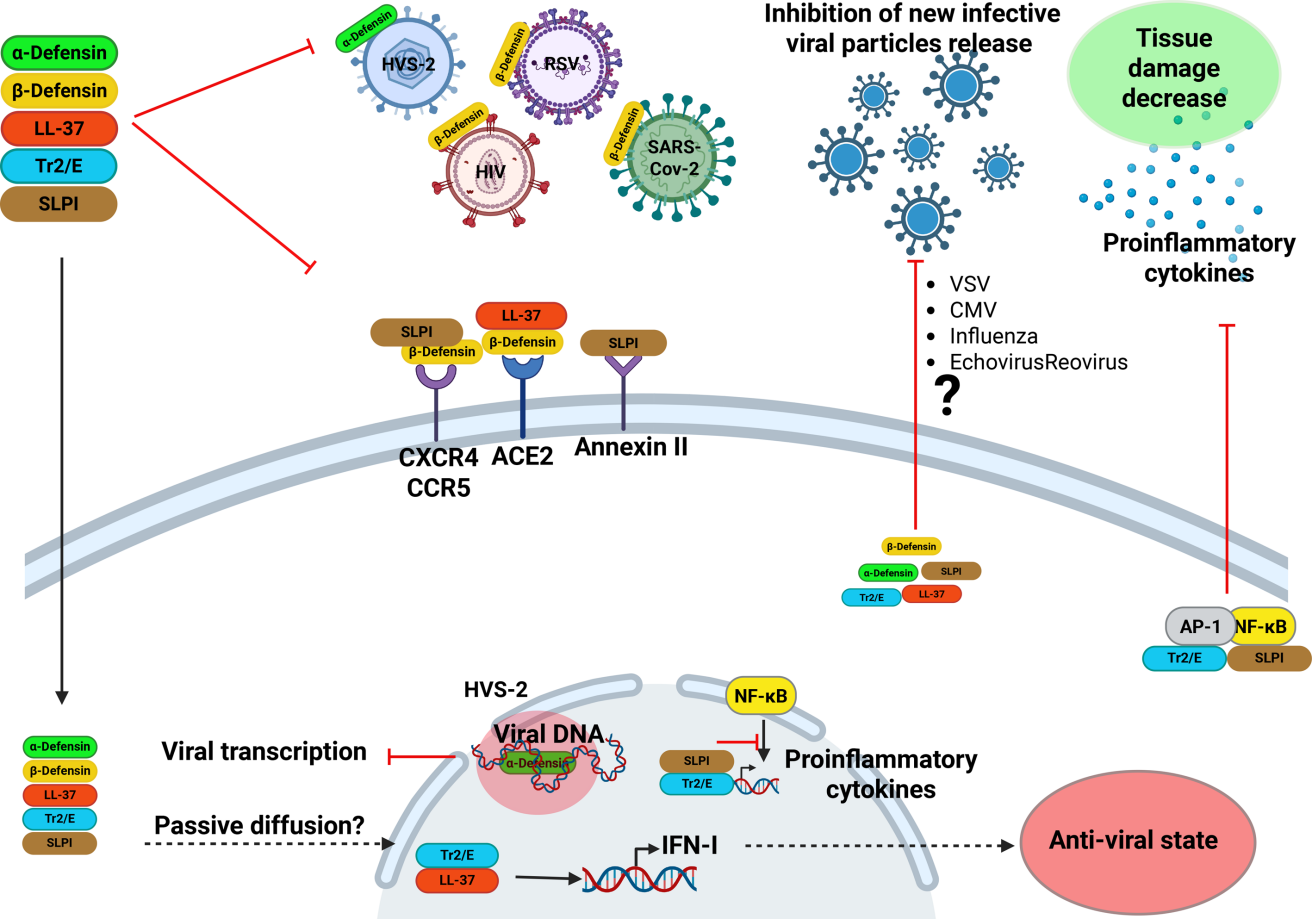
Effect of AMPs on some viral replicative steps. The main steps of the viral replication cycle are attachment, penetration, uncoating, replication, assembly and cell release. During the viral cycle, innate immunity mechanisms are triggered in the host cell, such as the production of IFN-I, establishing the antiviral state mediated by the innate immune response. During infection, tissue damage may also occur. AMPs can inhibit the binding of the virus to its cellular receptor, acting directly on the viral particle, or with its cellular receptor, causing the inhibition of the entry of this virus into the target cell. Regarding the replication of some DNA viruses, these peptides are capable of binding to viral DNA, inhibiting both the replication and transcription of viral protein. These peptides are capable of inhibiting the production of infectious viral particles to the extracellular environment; however, the mechanisms that impact these events have not been fully described, but they may act on the assembly of the viral particle or the exit of the virus. When the immune system is overactivated due to infection by some viruses, AMPs can act as immunomodulatory molecules, counteracting the production of some proinflammatory interleukins, protecting the host from tissue damage and aiding the survival of the infected host. Finally, the same immunomodulatory property of these peptides has been described to promote the expression of IFN-I.

AMPs exhibit antibacterial, antifungal and antiviral activities and possess immunomodulatory properties. Their expression and regulation differ amongst AMP types. Fig. 1 summarizes the antiviral mechanisms of AMPs, with a specific focus on their antiviral activities.

## Novel immunomodulatory mechanisms of AMPs as an antiviral effect

Previously, we described some antiviral mechanisms of AMPs. Nonetheless, the antiviral effect of these peptides extends beyond direct interactions with viral particles or cell receptors, as AMPs also possess immunomodulatory properties. Despite limited knowledge about their immunomodulatory roles, this mechanism relies on two intrinsic properties of AMPs: (1) their ability to undergo passive transport through membranes, translocating from the extracellular environment to the cytoplasm and nucleus, and (2) their ability to interact with DNA.

Studies have shown that some β-defensins, such as human beta-defensin (HBD)-1, are localized in the nucleus. Interestingly, in samples from normal skin of burned patients, the translocation of these peptides to the nucleus of keratinocytes was observed, suggesting their role in the regulation of gene expression [25,26]. Similarly, exogenous LL-37 is taken up by dendritic cells (DCs) and enters the nucleus in a dose-dependent manner. This nuclear localization of LL-37 correlates with phenotypic changes in DCs, including increased expression of the major histocompatibility complex molecule HLA-DR and the stimulatory molecule CD86, which could enhance the adaptive immune response under conditions of viral infection [27]. Furthermore, LL-37 has been detected in the nuclei of polymorphonuclear leukocytes under infectious conditions, which could be associated with its presence in neutrophil extracellular traps and its DNA-binding capacity, further amplifying its antimicrobial activity [28,30].

In contrast, the Tr2/E peptide translocates to the nucleus of epithelial cells, a process dependent on its amine terminal region. The antiviral activity of Tr2/E against HIV is associated with this translocation, which modulates the immune response by decreasing proinflammatory cytokines like IL-8 and TNF-α whilst increasing type I IFNs during viral infections [31,33].

SLPI and Tr2/E are AMP family members that possess WAP domains, enabling nuclear translocation and DNA binding. SLPI binds to p65 binding sites in the promoter regions of proinflammatory genes regulated by NF-κB, downregulating cytokines such as TNF-α, RANTES and IL-8 [34]. Similarly, Tr2/E reduces cytokine levels in epithelial cells, suggesting analogous effects. Tr2/E can also bind to the IL-6 promoter region to induce transcription [35].

**Fig. 2.**
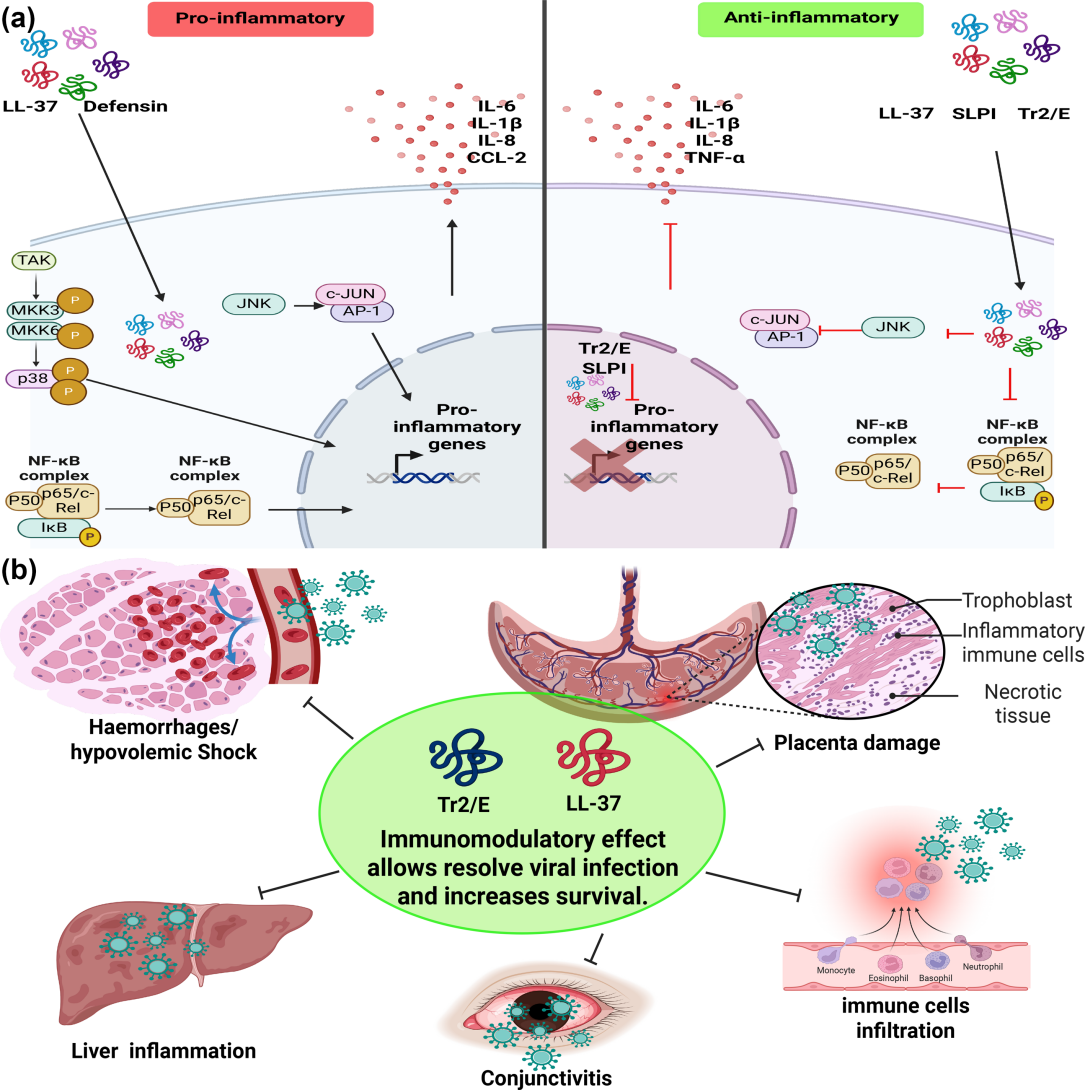
Immunomodulatory mechanisms of AMPs in *Flavivirus* infections. (a) AMPs regulate intracellular signalling pathways such as MAPK, cGAS-STING, NF-κB and cJUN-FOS, mediating the production of proinflammatory molecules. These mechanisms may help mitigate the systemic effects of exacerbated inflammation. (b) Infections by flaviviruses often trigger severe immune responses, causing tissue inflammation, haemorrhages and immune cell infiltration. Tr2/E and LL-37, with their anti-inflammatory properties, help maintain immunological and tissue homeostasis, potentially reducing severe clinical symptoms. Created using BioRender.com.

These findings underscore the antiviral potential of these peptides, which stems from their immunomodulatory characteristics. This opens new avenues for research into their antiviral properties (Figs 1 and 2).

## Antiviral effect of defensins

**Fig. 3.**
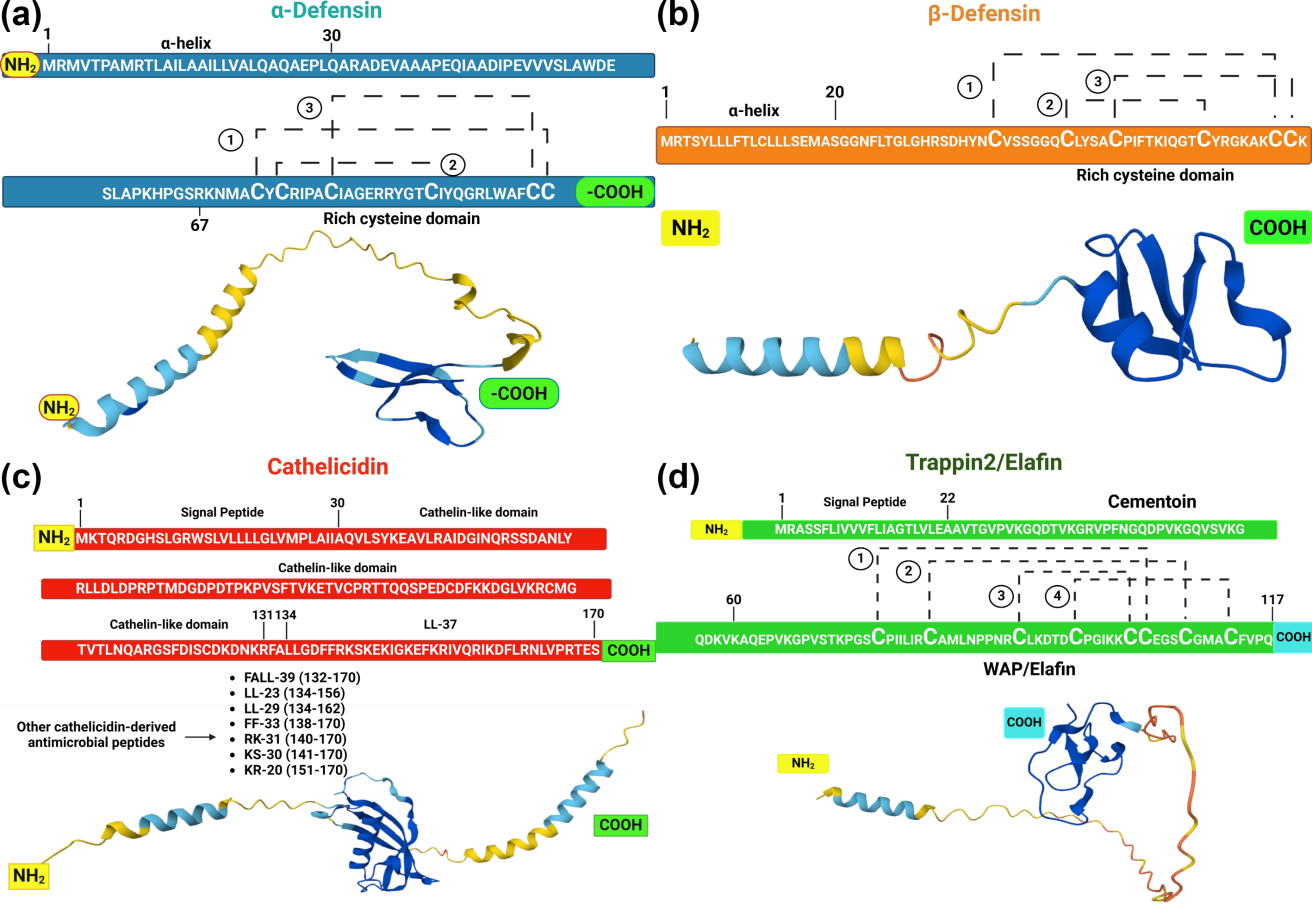
Schematic representation of selected AMPs. (a) α-Defensin sequence and structure: an α-helix in the amino-terminal region is followed by a bundle joining the carboxyl-terminal region, represented by β-folds. These folds form a core stabilized by three disulphide bridges mediated by cysteines C1–C6, C2–C4 and C3–C5. (b) β-Defensin sequence and structure: an α-helix in the amino-terminal region is similarly followed by a β-folded carboxyl-terminal region stabilized by three disulphide bridges mediated by cysteines C1–C5, C2–C4 and C3–C6. (c) Cathelicidin (LL-37) sequence and structure: composed of a signal peptide at the amino terminus, a cathelicidin-like domain and the LL-37 peptide at the carboxyl terminus. This peptide can also be processed into shorter AMPs. Residues composing these regions are indicated in parentheses. (d) Tr2/E peptide sequence and structure: consists of a signal peptide at the amino terminus, a cementoin domain and a WAP or elafin domain at the carboxyl terminus. The WAP domain is stabilized by four disulphide bridges mediated by cysteines, contributing to its antimicrobial characteristics. Created in BioRender.com.

Defensins, a prominent group of AMPs, are classified into three types: α, β and θ. Amongst these, α- and β-defensins are expressed in humans, whilst θ defensins are exclusive to non-human primates. This review focuses on α- and β-defensins, whose structural properties are depicted in Fig. 3. Defensins possess diverse biological activities, including antimicrobial action (against bacteria, fungi, parasites and viruses), immunomodulatory effects and antitumourigenic properties. Their expression is regulated by transcription factors such as NF-κB and STAT-1 [36,37].

α-Defensins are characterized by their 10–60 amino acid length and a six-cysteine motif that form three disulphide bridges at C1–C6, C2–C4 and C3–C5 positions, providing peptide stability. In contrast, β-defensins are 30–50 amino acids long and similarly enriched in cysteines, with three disulphide bridges at C1–C5, C2–C4 and C3–C6 positions, which also confer stability to the molecule [36,38, 39] (Fig. 3). The β-defensin group includes HBD-1, HBD-2, HBD-3, HBD-4, HBD-5 and HBD-6, whilst α-defensins include HNP1, HNP2, HNP3, HNP4, human alpha-defensin 5 (HD5) and human alpha-defensin 6 (HD6). These defensins are classified into specific members of their respective groups based on differences in charge, length and amino-terminal sequence identity changes [40,41].

Both groups of peptides are primarily expressed by immune system cells such as neutrophils, macrophages, natural killer cells, lymphocytes, DCs and platelets. However, they are also expressed in epithelial cells, including keratinocytes, pneumocytes and trophoblasts, as well as connective tissue cells such as fibroblasts [7,42]. The α-defensins, including HNP1, HNP2, HNP3, HNP4, HD5 and HD6, exhibit antiviral effects against viruses such as HSV-2 in a dose-dependent manner. They inhibit viral entry by directly binding to the viral surface glycoprotein gD and suppress the expression of early and late viral genes due to their DNA-binding capacity, attributed to their cationic properties [3,43]. Defensins such as HNP1 have demonstrated antiviral activity against various RNA viruses, including vesicular stomatitis virus, IVA, echovirus and reovirus, as well as DNA viruses such as cytomegalovirus [44].

Approximately 40 genes have been identified that encode different β-defensins [45,46]. Amongst these, the most studied are HBD-1, HBD-2, HBD-3, HBD-4, HBD-5 and HBD-6, which are primarily expressed in the immune system and epithelial cells. Some β-defensins possess antiviral effects, with HBD-2 being the most extensively studied. During HIV infection, HBD-2 exerts antiviral effects by binding to the cellular co-receptor CXCR4, which HIV uses for internalization, thereby reducing viral particle entry into cells [2]. Additionally, HBD-2 can interact with the receptor-binding domain (RBD) of the spike protein of SARS-CoV-2, preventing the interaction between the RBD and the hACE2 receptor, thus inhibiting infection [47]. It has also been observed that HBD-2 directly binds to viral particles, such as respiratory syncytial virus (RSV), destabilizing the virus and compromising the integrity of its virion structure [48] (Fig. 1).

## Cathelicidin (LL-37) and viral infections

LL-37 is a small cationic peptide encoded by the *hCAP-18* gene. The promoter of this gene contains binding sites for transcription factors such as STAT-3, HIF-1, VDRE (vitamin D3) and C/EBP [49]. LL-37 is the sole member of this peptide family in humans. This peptide can weigh up to 18 kDa. It is synthesized as a zymogen, requiring proteolytic cleavage by neutrophil serine protease 3 to become active. This cleavage generates a 37-amino-acid peptide called LL-37 [50,51] (Fig. 3). LL-37 is mainly expressed by epithelial cells, such as keratinocytes, and immune cells. It has been described to possess antibacterial, immunoregulatory, chemotactic and angiogenic properties, with its antiviral activities gaining prominence in recent years [52].

Recent studies have demonstrated that LL-37 exhibits antiviral activity against SARS-CoV-2 by directly binding to the RBD of the spike protein, thereby inhibiting its interaction with the hACE2 receptor [53,54]. Additionally, exogenous treatment with LL-37 has been shown to inhibit the production of new infectious RSV particles in lung epithelial cells during RSV infection [55] (Fig. 1).

## Protease inhibitors and their novel antiviral properties

Whilst much of the research on AMPs has focused on defensins and LL-37, recent years have seen a surge in the study of novel molecules such as protease inhibitors, which hold promise as novel antiviral agents. Protease inhibitor peptides are grouped into a single family characterized by domains with cores formed by disulphide bridges. These domains, known as WAP domains, consist of 50 cysteine-enriched amino acids and primarily function to inhibit leucocyte serine proteases [15]. In addition to their immunoregulatory role, these peptides have demonstrated antimicrobial properties against bacteria, fungi and viruses [15,17,56] (Fig. 1).

The SLPI peptide contains two WAP domains, is composed of 107 amino acids with a molecular weight of 11.7 kDa and exhibits cationic properties. SLPI inhibits the activities of leucocyte elastase, cathepsin G and chymotrypsin and is primarily expressed in epithelial cells [15]. Its expression is regulated by transcription factors such as NF-κB and IRF-1, which bind to Interferon-stimulated response element (ISRE)-like sites [57]. SLPI demonstrates antiviral activity against HIV-1 in macrophages by competing for the cellular cofactor annexin II, which HIV uses for infection. Annexin II binds to extracellular proteins via the WAP domain of SLPI [15,58].

Tr2/E, also known as SKALP or pre-elafin, is another WAP-domain-containing peptide encoded by the peptidase inhibitor 3 gene. The promoter of this gene is regulated by transcription factors such as AP-1, NF-κB, NF-IL-6 and SPI [59]. Tr2/E is 116 amino acids long and consists of two main domains: a cementoin domain and a WAP domain. The cementoin domain has binding properties to extracellular matrix proteins, such as transglutaminase, enhancing protection against proteases that attack the extracellular matrix. This zymogen is activated by human tryptase in the extracellular medium, releasing a peptide called elafin, which contains the WAP domain, and retains cationic properties and enhanced activity [60] (Fig. 3).

Tr2/E is predominantly expressed in epithelial cells and some immune cells, including monocytes [15,60]. Beyond its protease inhibitory activity, Tr2/E exhibits chemotactic, immunomodulatory and antimicrobial properties, as well as antiviral activity. For instance, during HIV-1 infection, exogenous Tr2/E treatment inhibits transcytosis, a viral spread mechanism, and binds cell surface molecules to prevent HIV attachment. These mechanisms contribute to the Tr2/E antiviral effect during HIV infections [31,32]. Other studies analysing vaginal washes from sex workers identified high concentrations of Tr2/E in individuals resistant to HIV infection compared to those susceptible. Treatments using vaginal washes from the resistant group showed a reduced rate of HIV infection in epithelial cells in contrast to washes from the susceptible group [32,61].

Eleven polymorphisms in the Tr2/E promoter region have been identified through analysis of 112 African-American individuals. Some individuals exhibited loss of transcription factor binding sites, such as those for GATA-1 and AP-1, suggesting that the presence of these polymorphisms may influence susceptibility to or protection against viral infections [62].

Furthermore, modifications to the Tr2/E promoter allow interactions with non-canonical transcription factors such as Foxa3. Taken together, polymorphisms and epigenetic modifications are critical for differential expression across various tissues and individuals [63]. Another possible antiviral mechanism of Tr2/E may involve its anti-inflammatory properties, which contribute to the resolution of viral infections that often produce an exacerbated inflammatory immune response. This was demonstrated in a transgenic murine model expressing human Tr2/E, which survived infections by the encephalomyocarditis virus, a virus that is typically lethal [64]. Finally, endogenous Tr2/E expression is essential for the innate immune defence of epithelial cells against HSV-2 infection [33]. These findings underscore the importance of studying this novel molecule in virology, both for its immunomodulatory properties and its intrinsic antiviral activity, as summarized in Fig. 1.

## Arboviruses are susceptible to AMPs

Arboviruses are a group of viruses classified by their transmission vectors, primarily arthropods such as mosquitoes and ticks. The most representative families include Togaviridae, *Bunyaviridae*, *Reoviridae*, *Orthomyxoviridae* and *Flaviviridae* [65,66].

Human AMPs, such as LL-37, have demonstrated antiviral activity against arboviruses. For example, LL-37 exhibits antiviral effects against Rift Valley fever virus at the post-entry level in peptide-pretreated Vero cells [67]. Similarly, LL-37 inhibits the replication of Venezuelan equine encephalitis virus in various cell types, including BV2 murine microglial cells, U87MG human astroglioma cells and HMC3 human microglial cells [68].

Despite the limited information available on human AMPs and their antiviral activity, research on peptides from non-mammalian organisms has demonstrated their antiviral potential against certain arboviruses, including chikungunya virus (CHIKV). CHIKV, an arbovirus belonging to the Togaviridae family, is a significant human pathogen transmitted by *Aedes* mosquitoes [69,70]. However, studies specifically focusing on the antiviral activity of human AMPs against CHIKV are scarce. Notably, an amphibian-derived peptide known as figainin-2 has been shown to exhibit antiviral effects against both CHIKV and yellow fever virus in HuH7-infected cells [71,72]. Furthermore, the mosquito *Ae. aegypti* responds to CHIKV infection by expressing defensins A and C. These defensins are thought to play a critical role in the mosquito’s immune response, maintaining a homeostatic state whilst harbouring the virus, thus highlighting their antiviral properties [73].

Whilst there is limited information on human AMPs against arboviruses, the therapeutic potential of these peptides is significant. Given the high human incidence and global impact of the *Flaviviridae* family, this review emphasizes the importance of studying human AMPs in the context of orthoflaviviral infections.

## Role of AMPs during orthoflaviviral infections

The role of AMPs during orthoflaviviral infections has gained attention over the past decade. Whilst studies have described the antiviral effects of synthetic peptides and peptides from plants, insects or poisonous animals against flaviviruses [72,74,78], research on human AMPs remains scarce.

This section focuses on the existing evidence regarding the antiviral roles of human defensins and LL-37 during orthoflaviviral infections, highlighting their potential as therapeutic agents, even though there are no reports to date regarding the role of Tr2/E in orthoflaviviral infections. However, preliminary data from our research group indicate that Tr2/E is produced by human keratinocytes in response to ZIKV infection. Also, Tr2/E inhibits ZIKV replication by reducing the release of infectious viral particles (unpublished data).

## DENV

DENV belongs to the *Flaviviridae* family and has four known serotypes (1–4). It is transmitted through the bite of vector mosquitoes, including *Ae. aegypti*, *A. albopictus* and *Aedes polynesiensis*. The virus typically enters mainly through the skin and can spread to various organs such as the liver, kidneys, spleen and lymph nodes. Clinical manifestations range from mild fever, headache and muscle and joint pain to more severe conditions like dengue haemorrhagic fever. Haemorrhagic fever is characterized by high fever, hepatomegaly, circulatory failure, rash and haemorrhagic manifestations. In severe cases, significant plasma leakage can lead to complete circulatory shock and even death.

Due to the biological complexity of orthoflaviviruses, including DENV, ZIKV and WNV, the development of effective vaccines and drug applications is challenging. AMPs have emerged as a promising alternative, as evidence supports their role in combating *Orthoflavivirus* infections.

**Fig. 4.**
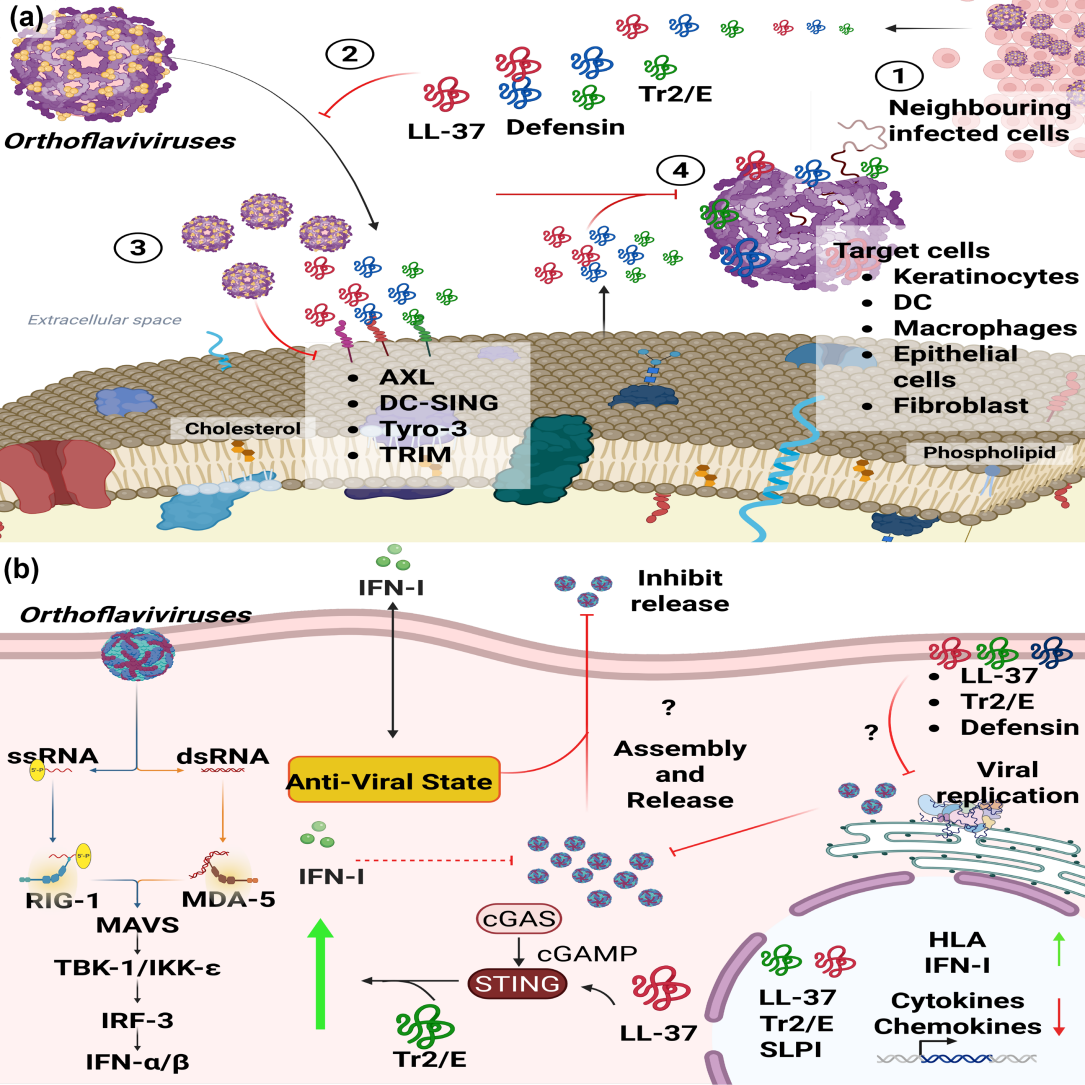
AMP anti-flavivirus mechanisms. (a) Representative diagram of extracellular mechanisms of AMPs against flaviviruses. (1) Infected cells respond to infection by producing defensins, LL-37 and Tr2/E, which are secreted into the medium to exert their effects. (2) These peptides bind directly to viral particles, preventing interaction with cellular receptors. (3) AMPs anchor to cell surfaces by binding to viral receptors, thereby blocking viral entry into cells. (4) AMPs interact with viral particles in the extracellular medium, destabilizing them and causing the rupture of the viral envelope. (b) Intracellular mechanisms of action. AMPs are readily taken up by host cells, either into the cytoplasm or nucleus, through passive diffusion. Inside cells, AMPs trigger immune responses mediated primarily by type I IFNs, proinflammatory cytokines and chemokines. Created using BioRender.com*.*

Studies have demonstrated the early antiviral response in skin cell models using DENV-infected primary fibroblasts. These cells produce HBD-2 and HBD-5 in response to infection [79]. Similarly, human keratinocytes infected with DENV-2 produce HBD-2, HBD-3 and LL-37. In both cellular and mouse models, exogenous treatment with these peptides showed a dose-dependent antiviral effect by inhibiting the production of new infectious viral particles [80]. Additionally, monocytes and neutrophils infected with DENV responded by producing HBD-1 and LL-37 as early as 6 h post-infection [81]. Pre-incubation of Vero E6 cells with LL-37 reduced viral RNA production and NS1 protein levels in the supernatant, suggesting that LL-37 interacts with the DENV E protein, which facilitates viral entry into target cells [82] (Fig. 4).

## ZIKV

ZIKV, another member of the *Flaviviridae* family, is transmitted not only by *Aedes* mosquitoes but also through blood transfusions, sexual contact and vertical transmission. The virus exhibits tropism for a wide range of tissues, including the skin, liver, kidneys, heart, spleen, placenta, testicles and brain. Clinical manifestations include rash, conjunctivitis, muscle and joint pain, fever and severe complications such as testicular damage leading to infertility and neurological conditions like Guillain–Barré syndrome and congenital Zika syndrome, which is characterized by microcephaly and delayed neuronal development in newborns.

Research into the antiviral role of AMPs during ZIKV infections remains limited; however, recent studies have provided valuable insights. For example, in 2020, Li *et al*. reported that epithelial cells infected with ZIKV secrete exosomes containing mRNA coding for α-defensin 1B (DEFA1B). These exosomes exhibit antiviral activity by inhibiting viral replication in cells treated with DEFA1B-inducing exosomes [83]. Moreover, exogenous treatment with LL-37 inhibited ZIKV replication in infected macrophages in a dose-dependent manner [84] (Fig. 4). Further investigations have demonstrated that cathelicidin peptides can be processed into smaller peptides with antiviral properties. Peptides derived from human and bovine cathelicidin, such as GF-17 and BMAP-18, showed antiviral activity in pre- and post-treatment of Vero cells and human foetal astrocytes. These peptides activate the type I IFN response during exogenous treatment, suggesting that their antiviral effect is mediated through the IFN response and direct inactivation of viral particles [85] (Fig. 4).

## WNV

The antiviral role of human AMPs during infections by other orthoflaviviruses is less extensively studied; however, recent advancements have highlighted the potential of these molecules. For instance, exogenous treatment with LL-37 in human primary keratinocytes inhibited WNV replication, suggesting a direct interaction with the viral particle. In contrast, keratinocytes treated with HBD-3 did not inhibit WNV replication. Nevertheless, both peptides demonstrated immunomodulatory properties during co-stimulation with poly I:C in human primary keratinocytes, enhancing the antiviral response [86] (Fig. 4).

## Applications as therapeutic targets

Managing *Orthoflavivirus* infections remains challenging due to the complexity of their biology, which hampers the development of effective vaccines and treatments. In this section, we summarize the most relevant information on the application of AMPs as safe prophylactics or treatments. AMPs exhibit low cytotoxicity, no resistance development and high efficacy against viral infections, making them promising candidates for resolving *Orthoflavivirus* infections.

Severe clinical cases of DENV and ZIKV infections are often linked to a ‘cytokine storm’ caused by an exaggerated proinflammatory immune response. The immunoregulatory properties of AMPs can help mitigate this, allowing the host to mount a robust immune response without compromising tissue integrity (Fig. 2).

Considering that *Orthoflavivirus* infections transmitted via mosquito bites begin in the skin, exogenous administration of peptides like defensins, LL-37 and Tr2/E may reduce systemic viral dissemination. For instance, pharmaceutical formulations containing AMPs in topical gels have been proposed for treating genital herpes in animal models [3]. This approach could inspire the development of AMP-based formulations for cutaneous application, either immediately after a mosquito bite or as a prophylactic measure in tropical regions where the vector is endemic.

ZIKV infection during pregnancy is well-documented to cause Zika-associated congenital syndrome and microcephaly in newborns, conditions strongly associated with significant placental tissue damage. This damage is characterized by elevated levels of proinflammatory cytokines and chemokines and infiltration of immune system cells [87,88]. Placental trophoblasts are known to produce higher levels of Tr2/E peptide than other AMPs, such as defensins like HBD-1 and HBD-2, during inflammatory stimuli [89]. This suggests that AMPs are crucial players in the antiviral response during ZIKV or DENV infections.

We propose that exogenous treatment with the Tr2/E peptide could serve as a potential therapeutic approach for ZIKV or DENV infections during pregnancy due to its immunoregulatory properties. Furthermore, studies have reported that exosomes loaded with the LL-37 peptide effectively inhibit ZIKV replication and dissemination to the placenta in murine models. This approach has been proposed as a treatment for placental damage to reduce the risk of abortions caused by DENV or congenital malformations caused by ZIKV. The viability and safety of such treatments have also been highlighted in these studies [90].

ZIKV can cross the placental barrier and the CNS through transcytosis [91,92]. Notably, treatments with the Tr2/E peptide have been shown to inhibit the transcytosis of viruses such as HIV [32]. This mechanism could contribute to the systemic antiviral effects of Tr2/E in preventing ZIKV dissemination. Additionally, LL-37-loaded exosomes have been demonstrated to reduce testicular damage and enhance sperm health in murine models, further supporting their therapeutic potential [90] (Fig. 2).

Another promising pharmaceutical option involves the use of AMP inducers. LL-37 expression has been reported to be upregulated by administering vitamin D and sodium butyrate, both of which are already available in safe pharmaceutical formulations for human use. According to the previously discussed AMP’s antiviral activity, these compounds can be employed as prophylactic treatments or during the course of DENV and ZIKV infections [84,93, 94] (Table 1).

**Table 1.** Potential therapeutic uses of AMPs during *Orthoflavivirus* infection

Therapeutic use	Peptide	Application method	Reported mechanisms	Reference
Prophylactic treatment (focused in high prevalence of mosquito vector areas).	LL-37Tr2/ESLPI	-Intravenous, loaded in exosomes.-Administration of commercial formulations of vitamin D and sodium butyrate.	-Promotion of AMP’s synthesis-Promotion of type I IFN response-Controlling viral dissemination	[31,33,84, 90, 93]
Treatment during progression on infected patients.	DefensinsLL-37Tr2/E	-Direct application on mucous membranes.-Intravenous, loaded in exosomes.	-Controlling viral dissemination into susceptible regions like testicle, placenta and nervous system.-Controlling inflammation in placenta to avoid congenital syndrome.-Controlling cytokine storm	[3,31,35, 90]

## Conclusion

The *Flaviviridae* family includes pathogens with high global incidence, capable of causing potentially fatal or neurodegenerative diseases. Currently, there are no safe and effective vaccines or pharmacological treatments to prevent infections by orthoflaviviruses such as DENV and ZIKV or to treat these infections during their progression. In this context, human AMPs have emerged as promising therapeutic targets. Amongst these, defensins and human cathelicidin are the most studied AMPs. However, novel molecules like Tr2/E have demonstrated significant antiviral and immunomodulatory capacities, offering potential for resolving viral infections caused by orthoflaviviruses such as ZIKV and DENV.

## Abbreviations

AMPs: antimicrobial peptides
CHIKV: chikungunya virus
CNS: central nervous system
DCs: dendritic cells
DEFA1B: α-defensin 1B
DENV: dengue virus
HBD: human beta-defensin
HIV: human immunodeficiency virus
HSV: herpes simplex virus
IVA: influenza virus
RBD: receptor-binding domain
RSV: respiratory syncytial virus
SLPI: secretory leucocyte protease inhibitor
TBEV: tick-borne encephalitis virus
Tr2/E: trappin-2/elafin
WAP: whey acidic protein
WNV: West Nile virus
ZIKV: Zika virus
